# Iron Regulation in Elderly Asian Elephants (*Elephas maximus*) Chronically Infected With *Mycobacterium tuberculosis*

**DOI:** 10.3389/fvets.2020.596379

**Published:** 2020-10-30

**Authors:** Maja Ruetten, Hanspeter W. Steinmetz, Markus Thiersch, Marja Kik, Lloyd Vaughan, Sandro Altamura, Martina U. Muckenthaler, Max Gassmann

**Affiliations:** ^1^PathoVet AG, Pathology Diagnostic Laboratory, Lindau, Switzerland; ^2^Tierpark Hellabrunn, München, Germany; ^3^Institute of Veterinary Physiology, and Center for Clinical Studies, Vetsuisse Faculty Zurich, and Zurich Center for Integrative Human Physiology (ZIHP), University of Zurich, Zurich, Switzerland; ^4^Pathology Division, Department of Biomedical Health Sciences, Veterinary Medicine, Utrecht University, Utrecht, Netherlands; ^5^Department of Pediatric Oncology, Hematology, Immunology and Pulmonology, Children's Hospital, Heidelberg University Medical Center, Heidelberg, Germany; ^6^Translational Lung Research Center Heidelberg (TLRC), German Center for Lung Research (DZL), University of Heidelberg, Heidelberg, Germany; ^7^Molecular Medicine Partnership Unit, University of Heidelberg, Heidelberg, Germany; ^8^Universidad Peruana Cayetano Heredia (UPCH), Lima, Peru

**Keywords:** nutritional immunity, anemia, hepcidin-ferroportin axis, secondary hemosiderosis, iron storage disease, interleukin-6, transferrin receptor, ferritin

## Abstract

Restriction of nutrients to pathogens (nutritional immunity) is a critical innate immune response mechanism that operates when pathogens such as *Mycobacterium tuberculosis* have the potential to evade humoral immunity. Tuberculosis is of growing concern for zoological collections worldwide and is well-illustrated by infections of Asian and African elephants, where tuberculosis is difficult to diagnose. Here, we investigated hematological parameters and iron deposition in liver, lung, and spleen of three Asian elephants (*Elephas maximus*) infected with *Mycobacterium tuberculosis*. For reference purposes, we analyzed tissue samples from control *M. tuberculosis*-negative elephants with and without evidence of inflammation and/or chronic disease. Molecular analyses of bacterial lesions of post mortally collected tissues confirmed *M. tuberculosis* infection in three elephants. DNA sequencing of the bacterial cultures demonstrated a single source of infection, most likely of human origin. In these elephants, we observed moderate microcytic anemia as well as liver (mild), lung (moderate) and spleen (severe) iron accumulation, the latter mainly occurring in macrophages. Macrophage iron sequestration in response to infection and inflammation is caused by inhibition of iron export via hepcidin-dependent and independent mechanisms. The hepatic mRNA levels of the iron-regulating hormone hepcidin were increased in only one control elephant suffering from chronic inflammation without mycobacterial infection. By contrast, all three tuberculosis-infected elephants showed low hepcidin mRNA levels in the liver and low serum hepcidin concentrations. In addition, hepatic ferroportin mRNA expression was high. This suggests that the hepcidin/ferroportin regulatory system aims to counteract iron restriction in splenic macrophages in *M. tuberculosis* infected elephants to provide iron for erythropoiesis and to limit iron availability for a pathogen that predominantly proliferates in macrophages. Tuberculosis infections appear to have lingered for more than 30 years in the three infected elephants, and decreased iron availability for mycobacterial proliferation may have forced the bacteria into a persistent, non-proliferative state. As a result, therapeutic iron substitution may not have been beneficial in these elephants, as this therapy may have enhanced progression of the infection.

## Introduction

Tuberculosis in zoological gardens is a major health concern, especially when it affects large land mammals, such as Asian or African elephants (1, 2). The causative agent of this disease is *Mycobacterium tuberculosis*, a facultative intracellular pathogen capable of surviving in an infected host with little or no replication (3). Once immune control weakens, bacterial growth can resume, and active tuberculosis develops. In elephants, disease monitoring over their long lives is problematic as potential sites of infection are often simply not accessible for sampling. Diagnostic tests of live animals are usually limited to analyses of bodily fluids, which although largely non-invasive for the elephant, are restricted in their ability to provide clinical insight (3). This is unfortunate as such insight would provide an improved understanding of host defenses to chronic mycobacterial infections and help improve assessment of the possible zoonotic risk, the latter a real risk due to the intimacy between elephants and caretakers or visitors of these highly valued and endangered species (4). This capacity of *M. tuberculosis* to persist in the host is also a major obstacle for tuberculosis treatment, since most antibiotics are inefficient at reaching and killing quiescent bacteria (5), thereby further complicating attempts to treat large animals.

Iron is an essential element for the growth and virulence of *M. tuberculosis* (6). Like most living organisms, this pathogen requires iron as a cofactor for vital biological processes (7). Infected hosts restrict iron access to extracellular pathogens by inhibiting iron export from either macrophages that turn over large amounts of iron by recycling senescent red blood cells or from duodenal enterocytes that are responsible for dietary iron absorption (8). Both processes require efficient iron export that is regulated by ferroportin, the only known iron exporter. Ferroportin protein expression at the cell surface of macrophages or enterocytes is controlled by the peptide hormone hepcidin. Hepcidin transcription is activated by inflammatory cytokines, such as interleukin-6 (IL6) during infections (9). In parallel, bacterial patterns (e.g., Gram positive and Gram negative cell wall components) are recognized by Toll-like receptors that control signaling pathways that repress ferroportin transcription in macrophages (10). As a consequence of hepcidin-dependent and hepcidin-independent mechanisms, iron is sequestered in macrophages during inflammatory states. This is an important host defense mechanism for which the term nutritional immunity has been coined (11, 12). Nutritional immunity restricts pathogen proliferation by limiting the pathogen's access to essential elements, such as iron.

Unfortunately, these innate immune mechanisms may be circumvented by hereditary iron storage disease (ISD), observed in exotic species maintained in zoological gardens (13). In this disease, mutations in hepcidin activators cause low hepcidin expression, resulting in high iron export from macrophages and low intracellular iron content as a consequence. While animals in the wild have evolved to flourish on diets with inherently low or high iron contents by utilizing physiological mechanisms to promote or inhibit iron uptake, animals in captivity, however, are provided with a standard diet across all animal species that may be inadequate for some of them. The pathomechanisms for increased iron deposits in exotic animal species is rather poorly understood. Several species kept in collections show at least minor iron deposits in their inner organs that can be associated with tissue damage (14–17). For example, browsing animals are believed to have fewer iron depositions when living in the wild than grazers. This might be because browsing animals take in more iron-chelating tannins with their diet, that is generally also low in vitamin C and iron (18, 19). Differences in the pathomechanisms of iron storage can be exemplified by the minah bird (*Gracula religiosa*), that in contrast to the chicken (*Gallus gallus domesticus*) expresses high levels of the divalent metal transporter 1 (DMT1) and ferroportin, thus suggesting excess dietary iron absorption (20). Another example is the black rhinoceros (*Diceros bicornis*) which carries a S88T mutation in the *HFE* gene, the most frequently mutated gene in human patients with the iron overload disorder, Hereditary Hemochromaotosis. The S88T mutation inhibits binding to transferrin receptor 1, and therefore may indeed be pathogenic (21, 22). Mostly, however, the presence of ISD is a reflection of a nutritional imbalance, with excessive dietary iron leading to deposits of iron in tissues.

Whether elephants and other exotic animals have a nutritional immune mechanism to restrict iron (or other nutrients) for bacterial proliferation is currently unknown. To address this question, we analyzed elephants with a chronic mycobacteria infection, revealing the presence of ISD. We describe the location of iron deposition in tissues occurring together with chronic mycobacterial infection. Compared to tuberculosis-free control animals, elephants chronically infected by *M. tuberculosis* showed reduced blood iron levels and decreased hemoglobin values as well as higher iron deposits in macrophages. We postulate that, in mycobacterial infected elephants, mechanisms are operational that deprive iron from microorganisms and inhibit bacterial proliferation as a nutritional immune defense of the body. Accordingly, we investigated the hepcidin/ferroportin regulatory system in elephants at necropsy which had been subjected to long-standing tuberculosis with acute and chronic inflammation.

## Materials and Methods

### Animals

Necropsy and analyses of the specimens presented in this report were performed within a diagnostic context, meaning no animals were euthanized for the purposes of this research project and ethical approval was not necessary. The privacy rights of the zoo were fully protected and the data obtained were anonymized.

Three female Asian elephants were euthanized due to severe geriatric health problems, such as weight loss, weakness and exercise intolerance. The elephants were originally imported as juveniles from Burma (AE 1), India (AE 2), and Thailand (AE 3) to Europe and were 52, 54, and 52 years old, respectively, when euthanized. All elephants were housed in a female group and managed under free contact. All elephants were suspected to be infected with *M. tuberculosis*. Characteristics of all elephants are shown in Table 1.

**Table 1:** Elephant characteristics and mRNA expression levels of different genes in liver samples.

**Animal**	**Age group**	**Diagnosis**	**Perl's spleen**	**TIS liver**	**HAMP**	**SLC40A1**	**IL6**	**NOS2**	**FTH1**	**Serum amyloid A-1**	**BMP6**	**TFRC**
AE 1	Adult	Tuberculosis	High	3	0.36	1.64	1.1	0.1	1.77	0.16	1	0.04
AE 2	Adult	Tuberculosis	High	4	0.56	0.46	0.3	4.26	0.45	2.46	0.8	0.02
AE 3	Adult	Tuberculosis	High	10	0.07	1.64	0.01	0.43	0.32	0.12	0.01	0.02
NL 1	Adult	Purulent pododermatitis	High	4	Excluded	^**^	^**^	^**^	^**^	^**^	^**^	^**^
NL 2	Adult	Arthrosis	High	3	3.54	1.1	2.1	0.14	1.97	1.4	1.7	3.5
NL 3	Juvenile	Exudative pneumonia	High	0	0.47	0.16	1.7	0.06	0.48	0.87	0.45	1.42
NL 4	Neonate	Trauma by mother	Low	0	1.08	nr	0.03	16.38	6.41	0.52	0.02	nr
NL 5	Adult	Polycystic kidney	Low	0	1.32	0.44	0.04	1.48	1.78	0.65	0.01	5
NL 6	Adult	Encephalitis	High	0	Excluded, autolytic	^**^	^**^	^**^	^**^	^**^	^**^	^**^

*The age group, final diagnoses, iron content measured by Perl's staining in spleen and liver are indicated. Total iron count (TIS) was calculated by the algorithm in Table 2. Relative mRNA expression in the liver (normalized against β-actin) of hepcidin (HAMP), ferroportin 1 (SLC40A1), interleukin 6 (IL6), inducible nitric oxide synthase (NOS2), ferritin heavy chain (FTH1), serum amyloid A1 (LOC100669763), bone morphogenetic protein 6 (BMP6), and transferrin receptor 1 (TFRC) were compared with the iron content in the spleen, semi-quantified histologically with Perl's staining. The animals were grouped into tuberculosis (AE1, AE2, AE3), acute inflammation (exsudative pneumonia, NL3), chronic inflammation (arthrosis, NL2), and no inflammation but trauma by mother (NL4) and polycystic kidney (NL6). NL1 and NL6 were excluded because of anomalous β -actin melting curve and severe autolysis, nr, no result*.

** *(anomalous β-actin melting curve)*.

Control tissues from an additional six Asian elephants (NL1–NL6) were kindly provided by the Utrecht University, Veterinary Medicine, Department of Biomedical Health Sciences, Pathology division for comparison. These tissues were derived from Asian elephants housed in different zoological facilities in the Netherlands with necropsies performed at Utrecht University, Veterinary Medicine, Department of Biomedical Health Sciences, Pathology Division (Table 1). These samples consisted of fixed histological slides and their corresponding deep-frozen liver tissues. All samples were examined, regardless of their original pathological diagnoses. Two elephants without any evidence of inflammation or chronic disease (NL4–NL5) were defined as negative controls for the purpose of qPCR analysis of mRNA levels of hepcidin (HAMP), ferroportin 1 (SLC40A1), interleukin 6 (IL6), inducible nitric oxide synthetase (NOS2), ferritin heavy chain (FTH1), serum amyloid A1 (LOC100669763), bone morphogenetic protein 6 (Bmp6), and transferrin receptor 1 (TFRC).

### Blood

Venous blood samples from elephants AE 1, AE 2, and AE 3 were collected into 1 ml EDTA-tubes to measure erythroid and leucocyte parameters, including total erythroid counts, packed cell volumes (PCV), hemoglobin concentrations, mean corpuscular volumes (MCV), mean corpuscular hemoglobin concentrations (MCHC), mean corpuscular hemoglobin (MCH) and total leukocyte and monocyte counts. Heparin coated tubes were used for collecting blood to measure serum iron and liver enzyme, alanine aminotransferase (ALT). Twenty microlitres of thawed blood plasma was used for hepcidin detection using the Hepcidin 25 (bioactive) HS Elisa^®^ (DRG Instruments GmbH, Marburg, Germany), as per the manufacturer's instructions and using standard controls. The absorbance of each well was determined by a microtiter plate reader (spectramax M2e^®^) at 450 ± 10 nm within 10 min after adding the stop solution. No hematological values were available from elephants NL1-6.

### Necropsy and Histology

The euthanized elephants AE 1, 2, and 3 were dissected immediately after death. Internal organs were measured with at least two small samples (1 cm^3^) collected from each organ and stored in either 4% buffered formalin or in sterile tubes (for subsequent microbiology and molecular investigations) by quickly freezing on dry ice followed by storage at −80°C. The samples for histology were fixed in 4% phosphate-buffered formalin for 24 h before processing. The specimens were dehydrated by an ascending alcohol series (70–95%), finally placed in xylol and embedded in paraffin. Sections of 2 μm thickness from the tissue samples were mounted on glass slides and stained with haematoxylin and eosin (HE), Ziehl-Neelsen (ZN) and Perl's using standard protocols. The hemosiderin depositions in the cytoplasm of macrophages in the spleen were graded from 0 to 3, grade 0 referring to no iron depositions and grade 3 representing a red pulp, diffusely filled with macrophages positive for iron staining. In order to obtain a semi-quantitative assessment, we adapted a previously published protocol used for grading the iron load in livers of humans at risk of hemochromatosis (23) according to the one modified for lemurs (24), and used this protocol to gain an objective overview of the amount and distribution of iron deposits. The total iron score (TIS) is divided into the (i) hepatic iron score, that contains iron depositions intracytoplasmatically in hepatocytes in the three different zones of liver lobules, (ii) the sinusoid iron score, summarizing iron depositions in endothelial cells and Kupffer cells distributed in the different zones, and (iii) the portal iron score, which focuses on iron granules in macrophages periportally or deposits intracytoplasmatically in bile epithelial cells. The grades range from 0 to a maximum of 60 (Table 2). All histological slides from NL 1-6 were screened to review the previous diagnoses and to score the iron depositions in liver and spleen using Perl's staining.

**Table 2:** Total Iron Score (TIS) grading scheme.

**Type of Iron**	**Periportal**	**Midzonal**	**Centrolobular**
**Hepatic Iron Sore**			
Absence of iron	0	0	0
Faint staining	3	3	3
Non-confluent granules	6	6	6
Confluent deposits	9	9	9
Large masses	12	12	12
**Sinusoidal Iron Score**			
Absence of Iron	0	0	0
Faint staining	1	1	1
Small granules	2	2	2
Large deposits	3	3	3
Clusters of overloaded cells	4	4	4
**Portal Iron Score**			
Abscence of Iron	0	–	–
Deposits in <1/3 of portal fields	1	–	–
Deposits in 1/3 of portal fields	2	–	–
Deposits of >1/3 of portal fields	3	–	–
Deposits in all portal fields	4	–	–

*Semi-quantitative analysis of iron depositions in the liver after Perl's staining, adapted from (23). TIS consisted of hepatic iron store, sinusoidal iron store and portal iron store with scores ranging from 0 to 60. – = not relevant*.

### Isolation of mRNA Coding for Selected Iron Regulatory Proteins

Levels of mRNA of hepcidin (HAMP, XM_003420761.2), ferroportin 1 (SLC40A1, XM_003406172.3), interleukin 6 (IL6, XM_003407083.3), inducible nitric oxide synthase (NOS2, XM_023556122.1), ferritin heavy chain (FTH1, XM_023559769.1), serum amyloid A-1 (LOC100669763, XM_003411983.3), bone morphogenetic protein 6 (BMP6, XM_003417847.3), transferrin receptor 1 (TFRC, XM_010592893.2), were measured by qPCR. Samples of 5–10 mg were taken from the deep frozen liver tissue, finely crushed in liquid nitrogen and total RNA was extracted using the RNA isolation kit (SV total RNA isolation system, Promega^®^, Madison WI, USA). mRNA was transcribed into cDNA using M-MLV Reverse Transcriptase (Promega^®^, Switzerland) and cDNA was adjusted to 5 ng/μl. Real time PCR was performed in the Applied Biosystems 7500 Fast Real Time PCR System (ThermoFisher Scientific, Switzerland) using 10 ng cDNA and the LightCycler^®^ 480 SYBR Green I Master (Roche, Switzerland). The primers for real time PCR were designed based on the published RNA sequences (emsemble Loxafr3.0, INSDC Assembly GCA_000001905.1, Jul 2009) of the African elephant (*Loxodonta africana*) following identification of homologous gene sequences to the target genes. The forward and reverse primer sequences used are summarized in Table 3. The amplification protocol started with an incubation at 50°C for 2 min followed by an incubation at 95°C for 2 min. 40 cycles at 94°C for 30 s and at 60°C for 40 s for quantification were followed by a melting curves for quality assurance (multiple peaks were exclusion criteria). The PCR products were separated on a native polyacrylamide gel to verify the correct PCR product size. The CT values were determined software-supported by identifying the linear range of amplification. The CT method was used to determine relative mRNA levels qPCR (25).

**Table 3:** Primer sequences used for qPCR.

**Target gene**	**Forward primer**	**Reverse primer**	**Gene ID**	**Official symbol**
Hepcidin (hamp 1)	CTCCTTCGCCTCTGGATCAC	TAAGACTCCCTTCCGAGCCA	XM_003420761.2	HAMP
Ferroportin (Fpn) 1	GCAGGAGAAGACAGAAGCAAA	CGAAATGAAACCACAGCCGA	XM_003406172.3	SLC40A1
IL-6	TTCCACAGATGACAGAAGAAGATGG	TTGAAACTCCGAAAGACCAGTGA	XM_003407083.2	IL6
iNOS	GGAAGATGCTGAGAGACGGAGG	AGGAATGTAGGGCTGTTGGTGAA	XM_023556122.1	NOS2
Ferritin heavy chain	CAGAACTACCATCAGGACTTGGA	CTTCAGAGCCACATCATCGC	XM_023559769.1	FTH1
SAA-1	ATGTTCTGCTCCTTGGTCCTG	TCAGCCCTCGTGTCTTCATCT	XM_003411983.3	LOC100669763
BMP-6	CTCCAGTGCCTCAGATTACAACA	GACATACTCGGGATTCATAAGGTGG	XM_003417847.3	BMP6
Transferrin receptor 1	ATTAGTGGTCAGTCTCTCTATCAGG	AAAGGGAAAGCAGCATCATCA	XM_010592893.2	TFRC
ß-Actin	CCCTCTTCCAACCTTCCTTCCT	GGTCCTTCVCCATGTCAACG	XM_010596269.1	ACTB

### Bacterial Isolation, Spoligotyping, and Multilocus Variable-Number of Tandem Repeat Analysis (MLVA) and Whole-Genome Sequencing

Small samples from the lung tissue, several lymph nodes (pulmonary, mesenterial, and mandibular), rhinal mucosa, sinusoids, retropharyngeal, gastric mucosa and synovia, as well as feces were collected in sterile tubes for culture. The cultured isolates were then spoligotyped and submitted for multilocus, variable-number of tandem repeat analysis (MLVA) and whole-genome sequencing, as described previously (26). The sequencing data have been deposited in the European Nucleotide Archive (EMBL-EBI) under the study ID PRJEB21800 (26).

## Results

### Clinical and Hematological Assessments of Elephants AE1–AE3

The physical conditions of elephants AE 1 to 3 deteriorated slowly over 2 years prior to euthanasia. Weight loss and severe arthrosis with reduced mobility and lameness were observed. All three elephants tested positively for infection with a member of *M. tuberculosis* complex with DDP^®^ VetTB Assay (Chembio Diagnostic Systems, New York, USA), PPD^®^ (purified protein derivatory skin test,) and Lionex^®^ (Lionex-Elisa, Lionex GmbH, Braunschweig, Germany). Nevertheless, repeated trunk washes were negative for *M. tuberculosis* in culture and PCR analysis. All three elephants had a moderate, normochrome, normocytic anemia, ranging in their packed cell volume (PCV) from 22.9 to 30% (mean 26.3%, ref^.^ 34.7–42%), had low hemoglobin concentrations, ranging from 5.2 to 9.5 g/dl (mean 8 g/dl, ref. 11–14.4 g/dl) and low erythrocyte numbers ranging from 1.6 to 2.4 10e^6^/μl (mean 2.0 10e^6^/μl, ref: 2.5–3.9 10e^6^/μl). All other values, such as mean corpuscular volumes (MCV), mean corpuscular hemoglobin concentration (MCHC) and mean corpuscular hemoglobin (MCH) were normal (Table 4). The liver enzymes, leukocyte counts and monocyte counts were in their normal ranges. Serum iron levels were low in two out of the three elephants (Table 4). As reference we used the database of veterinary records of “Species 360” (27). The findings of the moderate normochrome, normocytic anemia is comparable with the values described for anemia of chronic inflammation in humans (28). Hepcidin serum levels were measured using an ELISA specific for human hepcidin in elephants with elephants AE 1, AE 2, and AE 3 recorded to have 5.0, 7.15, and 4.0 ng/ml, respectively. These values were low compared to normal human hepcidin levels, which range between 11.4 and 21.8 ng/ml (29), and also compared to hepcidin levels in healthy dogs (16.6 ± 7.7 ng/ml) (30). No blood values were available for the control elephants, NL1–NL6.

**Table 4:** Blood parameters of elephant AE1, AE2, and AE3.

	**Elephant 1**	**Elephant 2**	**Elephant 3**	**mean**	**Ref.species 360**
PCV %	30	26	22.5	26.3	38 (34.7–41.3)
Hb g/μl	9.18	9.5	5.2	8	12.7 (11–14.4)
Ec 10^6^ /μl	2.1	2.4	1.6	2	3.2 (2.5–3.9)
MCV fl	142.9	108.3	108.3	119.8	118 (100–136)
MCH pg	43.7	39.6	52.34	45.2	41 (35–47)
MCHC g/dl	30.6	36.6	32.1	33.1	33.8 (30.2–37.5)
Leucocyte 10^3^/μl	16.2	19.3	19.2	18.2	18 (13–23)
Monocyte 10^9^/μl	3.36	2.22	2	2.5	6 (1.5–10.5)
ALT U/L	3	2	2	2.3	8
Serum iron μmol/l	8.3	7.6	19.2	11.7	11.9 (11.4–12.4)
Serum hepcidin ng/ml	5.0	7.15	4.0	5.4	–

*Individual values are shown, followed by the mean value and compared to the published normal reference values of Asian elephants in species 360 (2020): all three animals show a moderate normochrome, normocytic anemia with low Hb content*.

### Gross Pathological Observations of Elephants AE1–AE3

**Figure 1:**
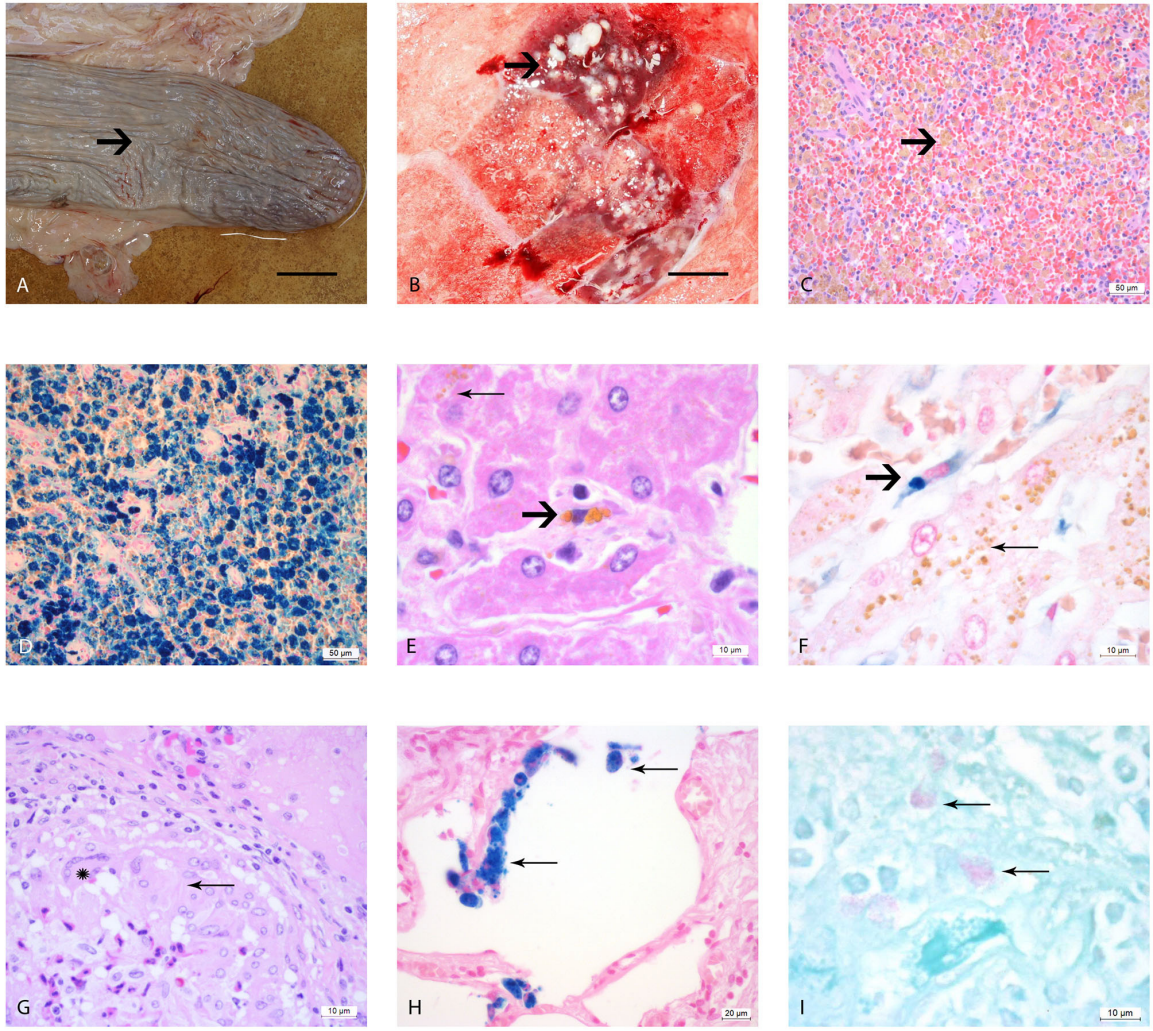
Macro- and microscopic analysis of the organs of elephants AE1-AE3 infected with *M. tuberculosis*. **(A)** Macroscopic image of the atrophic spleen with wrinkled capsule (arrow) and a flaccid consistency, bar = 10 cm; **(B)** Cut surface of right lung lobe, multiple caseous necroses (arrow) within the parenchyma and typical tubercle formations, measuring 1–2 cm in diameter, bar = 5 cm, **(C)** Histological image of the spleen, H&E staining, showing congested red pulp with multiple macrophages containing brown, granular pigment (hemosiderin) in their cytoplasm (arrow). **(D)** Histological image of the spleen with Perl's staining. The brown pigment (hemosiderin) shows a dark blue coloration. **(E)** Histological image of the liver, where the hepatocytes contain multiple small granular pigment in their cytoplasm (lipofuscin, wear and tear pigment, thin arrow). One resident spindeloid histiocytic cell in the middle of sinusoid shows coarser granular brown intracytoplasmic pigment (hemosiderin, bold arrow). **(F)** Histological image of the liver using Perl's staining, the histiocytic cells stain clearly blue (bold arrow) in contrast to the yellow pigment in the hepatocytes which retain their yellow color (thin arrow). **(G)** Histological image of a typical tubercle in the lung, forming a rim with epithelioid macrophages (thin arrow) and one multinucleated giant cell of Langhans'type (star). **(H)** Histological image of the lung using Perl's staining revealing several alveolar macrophages with a strong blue stained hemosiderin in their cytoplasm (thin arrows). **(I)** Histological image of the pulmonary lymph node using ZN staining, showing three macrophages with red staining plump acid fast rods intracytoplasmatically (thin arrows).

All three elephants (AE1–AE3) were in poor physical condition based on the degree of fat storage and muscle development. The spleens, measuring 150–176 cm in length, and 4–32 cm in width, had wrinkled surfaces, flaccid consistencies (Figure 1A), a lack of white pinpoints, and macroscopically visible lymph follicles, these observations being interpreted as atrophic lymphatic tissue. All three elephants had multiple granulomatous lesions in the lungs, with central caseous necroses (1–2 cm in diameter), surrounded by beige solid rims. The necroses found in all three elephants were occasionally calcified (Figure 1B). In a few lymph nodes, small (<5 mm) and solid granulomas were observed on the cut surfaces, but the general sizes of the lymph nodes were normal to small (1–2 cm). All elephants had severe erosive arthritis with cavernous lesions measuring up to 4 cm in diameter within the cartilage and extending into the underlying bony plate of all four carpal tarsal joints (data not shown). The kidneys of elephant AE1 were shrunken, firm and pale, measuring 23–34 cm, consistent with an elephant suffering chronic kidney failure. In comparison, the sizes of the kidneys of elephant AE2 were 38–45 cm and considered as normal. AE1 showed calcification in the stomach wall with small ulcerations and dystrophic calcifications within the lungs as sequelae to azotemia (uremic pneumonia), in addition to the granulomatous lesions. The parathyroid glands were enlarged. In addition to the tuberculoid lesions and arthritis, AE2 suffered from a severe pyometra. The uterus measured 125 × 122 cm and weighed 375 kg. The wall was severely thickened by fibrosis and hypertrophy of the smooth musculature and the lumen was filled with a purulent exudate. Lesions in AE3 were similar to those of AE1, but in addition exhibited chronic interstitial nephritis with fibrosis (size of the kidneys were 18–34 cm), uterine adenocarcinoma and several areas of ulcerative and hyperplastic dermatitis with fungal hyphae were observed.

### Histology of *M. tuberculosis* Infected (AE1–AE3) and Control Elephants (NL1–NL6)

The splenic white pulp of elephants AE1–AE3 was severely atrophic, with only very small lymph follicles with fading germinal centers and very small rims of periarterioles lymphocytic tissue (PALS) being left. The red pulp was filled with macrophages that contained intracytoplasmatic brown-yellow pigment (Figure 1C). These brown pigments stained an intense blue (strongly positive) with Perl's (Figure 1D). The iron loads in these spleens were graded as severe (Grade 3) according to the scheme we developed in this study. The liver lobules were mildly decreased in size, with macrophages, plasma cells and lymphocytes observed mildly infiltrating the periportal fields, with an additionally small increase of fibroblastic cells. The bile ducts were unremarkable. Several hepatocytes located pericentrally or midzonal were congested intracytoplasmatically with waxy brown, yellow pigments (lipofuscin), and was interpreted as a mild increase in hepatocyte turnover with hepatocellular degeneration (Figure 1E). The total iron scores varied from 3 to 10 in AE1, AE2, and AE3 (Table 1) and was interpreted as weak iron deposits in the liver (Figure 1F). All three elephants had a severe multifocal granulomatous pneumonia with typical tubercle formation with central caseous necrosis surrounded by multiple epithelioid macrophages, multinucleated giant cells and fibroblast formation (Figure 1G). Additionally, the number of alveolar macrophages in the alveolar spaces were increased and stained strongly positive with Perl's staining (Figure 1H). In contrast, the macrophages present in the granuloma formation showed no iron deposits. Small granulomas were seen in the pulmonary lymph nodes, consisting of mainly epithelioid macrophages and occasionally multinucleated giant cells of Langhans' type. A few macrophages in the lung, lymph node, tracheobronchial secretion and stomach mucosa contained small acid-fast rods intracytoplasmatically stained with ZN (Figure 1I).

Of the control elephants examined for comparison, NL2 and NL3 with high iron content in the spleen, were euthanized because of purulent pododermatitis, laminitis, arthrosis, exudative pneumonia and neutrophilic encephalitis caused by listeria infection. The only two elephants with low iron contents in their spleens died of severe trauma caused by the mother (NL 4) or by kidney cysts (NL 5). The total iron score of livers was low in all elephants with a score of “3” in elephant NL2, and “0” in elephants NL3, NL4, and NL5. The diagnoses and the iron content of the spleens and livers of the control elephants are summariz1ed in Table 1.

### Bacteriology

Eight *M. tuberculosis* isolates from AE1-3 were collected from lungs, sputum and stomachs and molecularly characterized by spoligotyping, as previously described (26). Spoligotyping revealed an identical profile for all the isolates, namely SIT276 (26). Further MLVA elucidated two divergent populations of bacteria and mixed infection in one elephant, suggesting either different transmission routes or prolonged infection over time (26). A total of eight *M. tuberculosis* colonies were subjected to whole-genome sequence analysis. Based on 99% sequence coverage and compared to the *M. tuberculosis* complex (MTBC) reference genome, sequence analysis revealed that all eight isolates were human-adapted strains belonging to MTBC Lineage 4 and sublineage L4.10, also referred to as principal genetic group 3. These findings suggest a single original species source of infection, namely human.

### mRNA Expression of Iron-Related Genes

We next determined hepatic mRNA levels of genes involved in iron regulation: hepcidin (HAMP), ferroportin 1 (SLC40A1), interleukin-6 (IL6), inducible nitric oxide synthase (NOS2), ferritin heavy chain (FTH1), serum amyloid A-1 (LOC100669763), bone morphogenetic protein 6 (BMP-6) and transferrin receptor 1 (TFRC). The mRNA levels were quantified from liver samples by qPCR from elephants AE1–AE3 and NL2–NL5. Elephants NL1 and NL6 were excluded because of autolysis and poor mRNA quality, respectively. Unfortunately, insufficient total RNA was available for completing qPCR analysis for the gene SLC40A1 in elephant NL4 (Table 1).

**Figure 2:**
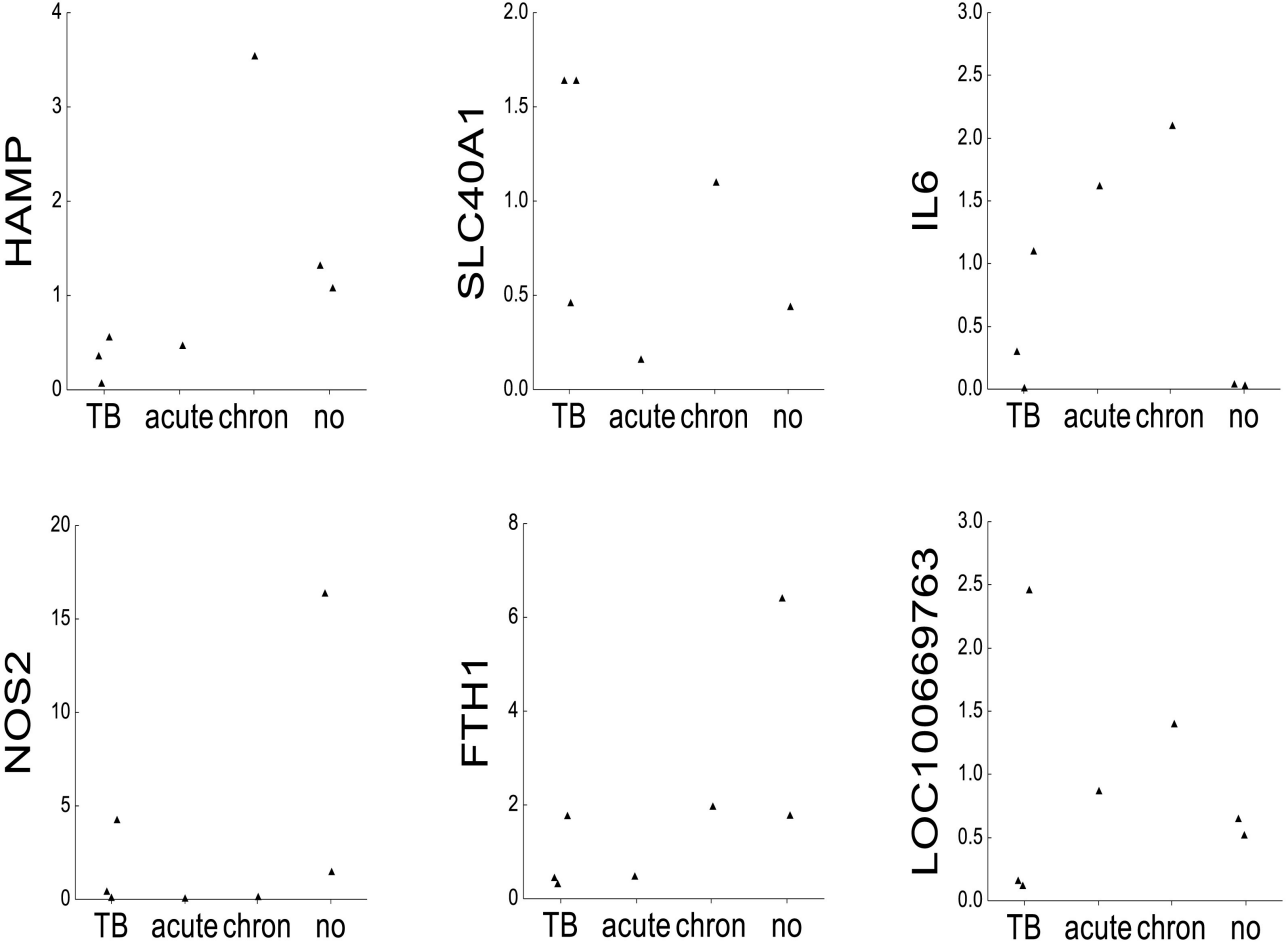
mRNA expression of iron-related genes. Dot plots representing on the x-axis 2^∧^ddCT mRNA levels normalized to β-actin of the following genes: hepcidin (HAMP), ferroportin 1 (SLC40A1), interleukin 6 (IL6), inducible nitric oxide synthase (NOS2), ferritin heavy chain (FTH1), serum amyloid A1 (LOC100669763), bone morphogenetic protein 6 (BMP6), and transferrin receptor 1 (TFRC) in seven elephants (AE1, AE2, AE3, NL2, NL3, NL4, and NL5). The animals were grouped after their diagnoses into tuberculosis (TB: AE1, AE2, AE3), acute inflammation (exsudative pneumonia, acute: NL3), chronic inflammation (arthrosis, chronic: NL2), and no inflammation (acute trauma by mother and polycstic kidney referred to as no: NL4 and NL5, respectively, shown on the y-axis).

For comparative purposes, the remaining elephants were arbitrarily divided into four groups (Figure 2): tuberculosis (TB: AE1-3), acute inflammation due to exudative pneumonia (acute: NL3), chronic inflammation due to arthrosis (chron: NL2), and no inflammation and death due to either trauma by mother or to polycystic kidneys (no: NL4 and NL5, respectively). The mRNA mean levels of HAMP in elephants with tuberculosis (AE1, AE2, AE3) were lower than the individual values in elephants with either acute (NL3), chronic (NL2), or without (NL4 und NL5) inflammation. Of note, the highest HAMP level was detected in NL2 suffering from chronic inflammation. In addition, SLC40A1 mRNA mean level in the elephants infected by tuberculosis was higher compared to the other elephants. Ferroportin protein could not be analyzed due to the lack of a specific antibody. Of note, FTH1 mRNA was only elevated in the elephant trampled by its mother (NL4). The distribution of TFRC, IL6 and BMP6 mRNA levels among the four groups appears to follow, to a given extent, the trend observed for hepcidin mRNA levels. Expression of NOS2 was elevated in the tuberculosis group but highest in the traumatized elephant (NL4). Serum amyloid A-1 (LOC100669763) expression levels were heterogeneous between the groups. Statistical analysis was not possible due to the low number of data points.

## Discussion

In this study, we showed for the first time that ISD exist also in elephants and in an exotic species is not only a matter of inadequate supply of a species-adapted diet but may be due to an underlying infectious disease, such as that caused by infection with *M. tuberculosis*. Three elderly Asian elephants AE1–AE3 were confirmed to be tuberculosis-infected. They showed moderate, normochromic and normocytic anemia with low Hb concentrations, low hepcidin levels and mildly reduced or normal serum iron levels that were accompanied by a massive sequestration of iron in the macrophages of the spleen and, less severe, in alveolar macrophages. No iron deposits were observed in hepatocytes, enterocytes, or macrophages lining the inflammatory wall of the tubercles that actually host tuberculosis bacteria intracellularly. Of note, iron distribution in elephants AE1 - 3 is different from that described in human hereditary hemochromatosis, a primary iron overload disease hallmarked by low hepcidin levels, in which macrophages are iron-spared (13, 31).

Elephants AE1–AE3 suffered from multiple pathologies as well as co-infections with *Listeria* that complicated the identification of the etiology of anemia. Chronic kidney disease was diagnosed in at least two of these elephants (AE1, AE3) that may have caused impaired erythropoietin production (32), possibly contributing to the mild anemia in these elephants. In addition, these geriatric elephants suffered from severe arthrosis known to lead to severe histiocytic inflammation and therefore increased IL-6 levels (33). Consistently, IL-6 levels and serum amyloid A1 (LOC100669763) levels were increased in elephants with chronic inflammation. Of note, FTH1 was highest in the animal group without inflammation. Since this group consists only of two individuals with only one of them showing an increased value, it is possible that the higher value may be an “outliner.” Chronic inflammation is hallmarked by hypoferremia and reduced hematopoiesis due to high hepcidin levels causing iron sequestration in macrophages (28). This mechanism leading to anemia through chronic inflammation is probably relevant in the control elephant NL2, suffering from severe arthrosis in the joints and high hepcidin expression. This may explain the elevated iron deposition in splenic macrophages of NL2. In contrast, elephants AE1–AE3 showed the lowest HAMP levels compared to all other groups. The low mRNA expression of HAMP was mirrored by serum hepcidin concentrations ranging between 4.0 and 7.15 ng/ml. Of note, serum levels of hepcidin in healthy Asian elephants have not been reported so far. Compared to healthy elderly humans showing a normal range of 11.4–21.8 ng/ml (29), and also compared to hepcidin levels in healthy dogs (16.6 ± 7.7 ng/mL) (30), it is justifiable to state that hepcidin serum concentrations of tuberculosis-infected AE1-3 were diminished. Therefore, hepcidin-mediated degradation of ferroportin cannot explain iron retention in macrophages, as observed in elephants AE1-3. In addition, macrophage iron sequestration is not due to low ferroportin transcription, as ferroportin mRNA levels were well-detectable. We therefore propose that the hepcidin/ferroportin regulatory system responds to the iron deficiency and mild anemia in tuberculosis-infected elephants to sustain iron availability for erythropoiesis. This suggests that a long-standing chronic infection with *M. tuberculosis* may not elicit an inflammatory response that inhibits iron export from macrophages. We speculate that elevated ferritin protein levels cause iron retention in macrophages of tuberculosis-infected elephants. These results are in line with previous publications (34, 35) showing that mycobacteria infections behave differently than other intra- and extracellular siderophilic infections, being not influenced by hepcidin ablation or by the hepcidin/ferroportin axis. This obviously requires further experimental investigation in subsequent studies. We would also like to point out that researchers dealing with such valuable animals should collect blood immediately prior to euthanasia and take more fresh tissue samples during necropsies. It is possible that the three elephants displayed tuberculosis symptoms at a younger age and, during this symptomatic time, the hepcidin concentration was elevated, as recently shown in tuberculosis patients in Tanzania (36).

What may be the benefit of iron accumulation in macrophages? On the one hand, it is anticipated that this will prevent the release of iron into the blood stream and thus inhibit bacterial (super-)infections and/or spreading of mycobacteria. On the other hand, mycobacteria proliferate in macrophages and thus iron may be a critical growth factor for their proliferation provided by iron retention. It is currently unclear whether mycobacteria can utilize iron from ferritin and whether iron retained in macrophages reaches the phagolysosome where mycobacteria reside. It is interesting to note that macrophages that are part of the mycobacteria-containing granuloma of the lung showed no iron deposits. On the other hand, iron is essential for the clearance of bacteria from tissues (37). This “oxidative burst” reaction requires the regeneration of reactive oxygen species and nitric oxide, processes that are critically dependent on iron (38). Iron accumulation in macrophages may contribute to a better immune defense against intracellular pathogens.

Finally, in humans, anemia of the elderly has been described. The etiology of this condition is unknown although it was previously thought that it is not related to chronic inflammation, chronic kidney disease or nutritional deficiencies (39). More recently, it was suggested that many factors such as nutritional insufficiency including Vitamin D and B12 deficiencies, low grade chronic inflammation of the liver, androgen deficiency, and sarcopenia may play a role (40). But whether these elderlies show an iron deposition in macrophages is not known. No similar condition has yet been shown to occur in geriatric elephants.

In conclusion, we showed that tuberculosis-affected Asian elephants develop ISD with anemia and low hepcidin levels but with iron accumulation predominantly in the spleen. We propose that iron sequestration in the elephant's spleen is a hepcidin-independent nutritional defense mechanism to deny iron access to mycobacteria to prevent their proliferation. The defense mechanism, however, trades suppression of bacterial growth for iron-deficiency anemia. From an animal management perspective, these results suggest that chronic infection in elephants and other exotic animal species kept in captivity with iron deficiency anemia should be excluded before supplementation of iron, which may trigger the revival of latent infections, such as *M. tuberculosis* or other pathogens. Additionally, we suggest excluding secondary diseases causing iron deposition in elephants and other animals before considering changing the diet to prevent dietary iron overload and related pathologies.

## Data Availability Statement

The raw data supporting the conclusions of this article will be made available by the authors, without undue reservation.

## Ethics Statement

Ethical review and approval was not required for the animal study because necropsy and analysis of the specimens was carried out within a diagnostic context, meaning that no animals were killed or additional tissue taken for the purposes of this research project and ethical approval was not necessary. Written informed consent was obtained from the owners for the participation of their animals in this study.

## Author Contributions

MR: collecting and analyzing the data, performing the necropsies, planning, and writing the manuscript. HS: collecting clinical data, performing diagnostic tests for mycobacteria infections, and writing clinical parts of the manuscript. MT: support with primer design and molecular data analyzing and writing parts of the manuscript. MK: providing elephant tissue, analyzing histological data of these animals, and writing parts of the manuscript. LV: support in analyzing molecular data and writing parts of the manuscript and correcting the english language of the manuscript in general. SA: providing valuable inputs in iron metabolism in humans and establishing hepcidin ELISA. MM: providing valuable expertise in human iron metabolism and co-writing the manuscript. MG: support of the research into iron metabolism in animals, providing valuable inputs and writing the manuscript with MR.

## Conflict of Interest

The authors declare that the research was conducted in the absence of any commercial or financial relationships that could be construed as a potential conflict of interest.
